# Supplementary material for: Histopathological Changes and Immune Response Profile in the Brains of Non-Human Primates Naturally Infected with Yellow Fever Virus
Source: Viruses. 2025 Mar 7;17(3):386. doi: 10.3390/v17030386 (PMC11946203; doi:10.3390/v17030386)

viruses MDPI

Histopathological Changes and Immune Response Profile in Brains of Non-Human Primates Naturally Infected by Yellow Fever Virus

Suzana Ribeiro de Melo Oliveira, Ermelinda do Rosário Moutinho da Cruz, Nelielma Garcia de Oliveira Prestes, Fábio Silva da Silva, Marialva Tereza Ferreira de Araújo, Orlando Pereira Amador Neto, Maria de Lourdes Gomes Lima, Bianca Nascimento de Alcântara, Daniel Damous Dias, Jorge Rodrigues de Sousa, Arnaldo Jorge Martins Filho, Livia Medeiros Neves Casseb and Daniele Barbosa de Almeida Medeiros

Supplementary material

Supplementary Table S1. Panel of the main histopathological findings of positive brain samples from NHPs infected with YFV from the meninges and parenchyma.

Group	ID	State	Year	Species	Lesion in Liver	IHC- YFV in Liver	Lesion in Brain	IHC- YFV in Brain
Aloutta sp. positive for YFV	476/15	Minas Gerais	2015	*Alouatta* sp.	manifest	detected	manifest	detected
	506/15	Goias	2015	*A. caraya*	manifest	detected	manifest	detected
	479/16	Goias	2016	*A. caraya*	manifest	detected	manifest	detected
	480/16	Goias	2016	*A. caraya*	manifest	detected	absent	undetected
	090/17	Minas Gerais	2017	*Alouatta* sp.	manifest	detected	manifest	detected
	273/17	Goias	2017	*A. caraya*	manifest	detected	manifest	detected
	397/17	Goias	2017	*A. caraya*	manifest	detected	manifest	detected
	568/17	Goias	2017	*A. caraya*	manifest	detected	manifest	detected
Callithrix sp. positive for YFV	573/16	Distrito Federal	2016	*C. penicillata*	manifest	detected	absent	undetected
	576/16	Distrito Federal	2016	*C. penicillata*	manifest	detected	absent	undetected
	686/16	Goias	2016	*C. jacchus*	manifest	detected	absent	undetected
	813/16	Rio de Janeiro	2016	*Callithrix* sp.	manifest	detected	absent	undetected
	815/16	Rio de Janeiro	2016	*Callithrix* sp.	manifest	detected	manifest	detected
	816/16	Rio de Janeiro	2016	*Callithrix* sp.	manifest	detected	absent	undetected
	907/16	Distrito Federal	2016	*C. penicillata*	manifest	detected	manifest	detected
	093/17	Minas Gerais	2017	*C. penicillata*	manifest	detected	manifest	detected
	097/17	Minas Gerais	2017	*Callithrix* sp.	manifest	detected	absent	undetected
	098/17	Minas Gerais	2017	*Callithrix* sp.	manifest	detected	absent	undetected
	100/17	Minas Gerais	2017	*Callithrix* sp.	manifest	detected	manifest	detected
	103/17	Minas Gerais	2017	*Callithrix* sp.	manifest	detected	manifest	detected
	278/17	Goias	2017	*C. penicillata*	manifest	detected	absent	undetected
	572/17	Goias	2017	*C. penicillata*	manifest	detected	absent	undetected
Negative Control	098/14	Goias	2014	*C. penicillata*	absent	undetected	absent	undetected
	077/16	Distrito Federal	2016	*Callithrix* sp.	absent	undetected	absent	undetected
	187/16	Rio Grande do Norte	2016	*C. jacchus*	absent	undetected	absent	undetected

Supplementary Table S2. Antibodies used in Immunohistochemistry assay.

Action	Biomarkers	Profile	Antibody	Animal origin	Code	Manufacturer
Pro-inflammatory	TNF-α	Th1	mAb	Rabbit	Ab6671	Abcam
	IL-1β	Th1	mAb	Rabbit	Ab9722	Abcam
	IFN-γ	Th1	pAb	Rabbit	Orb10878	Biobyt
	IL-12	Th1	mAb	Rabbit	Ab131039	Abcam
Anti-inflammatory	IFN-β	Modulator	pAb	Rabbit	Ab190211	Abcam
	TGF-β	Immunoregulation	mAb	Mouse	Ab190503	Abcam
	IL-4	Th2	pAb	Rabbit	Ab9622	Abcam
	IL-10	Th2	pAb	Rabbit	Ab98181	Abcam
	IL-13	Th2	pAb	Rabbit	Ab9576	Abcam
Cell Death	Casp 3	Apoptosis	pAb	Rabbit	Ab4051	Abcam

Legend: mAb – monoclonal antibody; pAb – policlonal antibody.

Supplementary Table S3. Spearman correlation between biomarkers whose p-values were significant for the NHP species.

Group	Variable Pair	Area	Correlation Coefficient (ρ)	Correlation Level	P-value	Statistical Significance
Alouatta	YFVAg vs IL-13[a]	M	-0.88191710	Strong (-)	0.008627408	significant
	YFVAg vs TGF-β[p]	P	0.81791288	Strong (+)	0.02457070	significant
	YFVAg vs TNF-α[a]	PV	-0.92418682	Strong (-)	0.002917058	Moderate significant
	IL-12[a] vs. TGF-β[p]	PV	-0.77951196	Strong (-)	0.03877851	significant
	TNF-α[a] vs. Casp3	P	-0.82997512	Strong (-)	0.02084423	significant
Callithrix	YFVAg vs IL-10	M	1	Strong (+)	0.00000000	very significant
	IL-4[a] vs. INF-γ[p]	PV	0.91666667	Strong (+)	0.02851396	significant
	IL-4[a] vs. IL-1β[a]	PV	0.91287093	Strong (+)	0.03046629	significant
	IL-4[a] vs. IL-13[a]	PV	0.9128709	Strong (+)	0.03046629	significant
	IL-1β vs. INF-α[p]	PV	0.91287093	Strong (+)	0.03046629	significant
	TNF-α[p] vs. IL-10[a]	PV	0.91666667	Strong (+)	0.02851396	significant
	TNF-α[p] vs. IL-12[p]	P	-0.4325905	Moderate (-)	0.005207859	very significant
	TNF-α[p] vs. IL-13[a]	P	0.91666667	Strong (+)	0.02851396	significant
	IL-13[a] vs. IFN-α[p]	M	-0.8838835	Strong (-)	0.04666188	significant
	IL-13 vs. IFN-β	M	-0.8838835	Strong (-)	0.04666188	significant

Legend: p = Pro-inflammatory cytokines; a = Anti-inflammatory cytokines: M = meninges; PC = parenchyma; PV = perivascular; * = significant; ** = very significant; *** = extremely significant.

Supplementary Table S4. Frequency of Hepatic lesion per NHP species. Data related to Figure 1B.

Type of lesion	Lesions in liver	*Alouatta* sp.		*Calithrix* sp.		p-Value
		**Individuals /Total**	**%**	**Individuals /Total**	**%**	
Degeneration	Tumefaction	08/ago	100%	14/14	100%	Not significant
	Steatosis[1]	06/ago	75%	mai/14	35.7%	Not significant
	Cholestasis	02/ago	25%	jun/14	42.9%	Not significant
Vessel injuries	Congestion[2]	04/ago	50%	mai/14	35.7%	Not significant
	Inflammation	06/ago	75%	nov/14	78.6%	Not significant
	Hemorrhage	01/ago	12.5%	mar/14	21.4%	Not significant
Cell death	Apoptosis[3]	02/ago	25%	jun/14	42.9%	Not significant
	Necrosis	08/ago	100%	ago/14	57.1%	Not significant
Regeneration	Regeneration[4]	03/ago	37.5%	mai/14	35.7%	Not significant
Tissue preservation	Autolysis	0/8	0%	0/14	0%	Not significant

Legend: individual that showed 1 = macrovacuolar and/or microvacuolar steatosis; 2 = vascular and or sinusoidal; 3 = Councilman bodies; 4 = Kupffer cell hyperplasia and/or hypertrophy; * not included in the graphic.

Supplementary Table S5. Frequency of lesion in the CNS per NHP species. From 22 NHPs analyzed, only 7/8 *Alouatta* sp. and 5/14 *Calithrix* sp. showed expressive damage in their CNS and had YFV antigen detected by IHC. This data is related to Figure 2B.

Type of lesion	Lesions in liver	***Alouatta* sp.**		***Calithrix* sp.**		p-Value
		**Individuals /Total**	**%**	**Individuals /Total**	**%**	
Degeneration	Edema	07/jul	100%	05/mai	100%	Not significant
	Neuronal degeneration	07/jul	100%	05/mai	100%	Not significant
Vessel injuries	Congestion	06/jul	85.7%	06/mai	100%	Not significant
	Inflammatory infiltrate[1]	02/jul	28.7%	03/mai	60%	Not significant
	Perivascular Edema	07/jul	100%	05/mai	100%	Not significant
	Hemorrhage[3]	02/jul	28.7%	03/mai	60%	Not significant
Cell death	Apoptosis/Necrosis	07/jul	100%	05/mai	100%	Not significant
Regeneration	Neurophagia	07/jul	100%	05/mai	100%	Not significant
Tissue conservation*	Autolysis	0/7	0%	0/5	0%	Not significant

Legend: individual that showed 1 = in meninges and/or vessel in parenchyma; 2 = in meninges and/or parenchyma; 3 = in meninges and/or vessel in parenchyma; 4 = Kupffer cell hyperplasia and/or hypertrophy.

Supplementary Table S6. Quantification tables of the main lesions found in the meningeal area.

Group	Monkey ID	Lesions in Meninges			
		**Congestion***	**Interstitial Edema***	**Hemorrhage***	**Inflammatory cells***
Alouatta sp. (n=7)	476/15	11	0	0	0
	506/15	0	0	0	0
	479/16	0	0	0	0
	090/17	0	0	0	0
	273/17	0	0	0	0
	397/17	11.6	47.6	2	0
	568/17	11.3	43.6	0	1.3
	Median	0	0	0	0
	Average	4.84	13.03	0.29	0.19
	IQR	11335	26.835	0	0
Callitrhix sp. (n=5)	815/16	12.6	11.3	0	1.3
	907/16	1.6	2.3	2	0
	093/17	15.6	18.3	27	1.6
	100/17	25	1.6	1.6	2.3
	103/17	12	22	0	0
	Median	12.67	18.33	0	0
	Average	13.36	11.10	6.12	1.04
	IQR	11.67	9	1.67	1

* The data represents the mean quantification of lesions across three randomly selected microscopic fields.

**Wilcoxon rank sum test with continuity correction comparing *Alouatta* sp. vs *Callithrix* sp. (Mann-Whitney test)**			
**Lesion**	**W**	**p-value**	**Statistical Significance**
Congestion	24.5	1	Not significant
Interticial edema	10	0.2385	Not significant
Perivascular edema	12	0.4085	Not significant
Hemorrhage	10.5	0.1046	Not significant
Inflammatory	10.5	0.1032	Not significant
**Hypotheses**			
**H0:** There is no significant difference between the groups ($p > 0.05$)			
**H1:** There is significant difference between the groups ($p < 0.05$)			

Supplementary Table S7. Quantification of the main lesions found in the parenchyma region.

Group	Monkey ID	Lesions in Parenchyma			
		**Cell death (necrosis/apoptosis)***	**Degeneration ***	**Neuronophagia ***	**Satellitosis***
Alouatta sp.	476/15	17.1	2.6	3	2
	506/15	24.3	7.3	9.3	2,67
	479/16	18	6	5	5
	090/17	26.6	2	5.6	5,67
	273/17	16.6	0.6	4	4
	397/17	16	2	6.3	5,33
	568/17	14.6	2.6	5.3	5,33
	Median	24.3	4	5	5
	Average	19.03	3.3	5.5	-
	IQR	27.5	3.7	1.14	1.995
Callitrhix sp.	815/16	20.3	2	1.3	1,33
	907/16	23.6	3	11.3	11,33
	093/17	19.3	6	3.6	7
	100/17	18.6	1.6	1.6	4
	103/17	25	0.3	7	3,67
		58.33	3.66	4	4
	Average	21.36	2.58	4.96	5.47
		4	2.33	1.33	3.33

* The data represents the mean quantification of lesions across three randomly selected microscopic fields.

**Wilcoxon rank sum test with continuity correction comparing *Alouatta* sp. vs *Callithrix* sp. (Mann-Whitney test)**			
**Lesion**	**W**	**p-value**	**Statistical Significance**
Cell death	5	0.05132	Not significant
Satelitose	24.5	1	Not significant
Degeneration	21.5	0.5677	Not significant
Neurophagy	24.5	1	Not significant
**Hypotheses**			
**H0:** There is no significant difference between the groups ($p > 0.05$)			
**H1:** There is significant difference between the groups ($p < 0.05$)			

Supplementary Table S8. Quantification table of the main lesions found in the perivascular region.

Group	Monkey ID	Congestion*	Satellitosis*	Lesions in Perivascular area			
				Cell death*	Perivascular Edema*	Hemorrhage*	Inflammatory cells*
Alouatta sp. (n=7)	476/15	6.3	1,67	1,33	1.3	0	0
	506/15	13	2,33	14,67	10	0	0
	090/17	0.6	1,33	1,33	2.6	31.3	1.3
	273/17	0	2	23,67	11.6	0	0
	397/17	15.6	3,33	5,67	4.6	2.6	0
	479/16	8.3	3	38	1	0	0
	568/17	23	2,33	51,33	9	0	9.3
	Median	8.33	2.33	14.67	2.67	0	0
	Average	9.54	2.29	19.43	5.73	4.84	1.51
	IQR	10.83	0.83	27.33	8.5	0	2.5
Callitrhix sp. (n=5)	815/16	1.3	2,33	50,67	4	0	0
	907/16	6.3	1,33	17,67	10.3	2.3	0
	093/17	6	5	30,33	18.3	10	0
	100/17	14.6	5,33	16	7.3	8	3.3
	103/17	0	3,33	48	3.5	0	0
	Median	6	3.33	30.33	7.33	0	0
	Average	5.64	3.53	32.53	8.68	4.06	0.66
	IQR	5	2.33	30.33	6.33	0	0

* The data represents the mean quantification of lesions across three randomly selected microscopic fields.

Wilcoxon rank sum test with continuity correction comparing Alouatta sp. vs Callithrix sp (Mann-Whitney test)			
**Lesion**	**W**	**p-value**	**Statistical Significance**
Congestion	23	0.4152	Not significant
Satelitose	9	0.1915	Not significant
Cell death	10	0.2548	Not significant
Perivascular edema	11	0.329	Not significant
Hemorrhage	17	1	Not significant
Inflammatory	20	0.6991	Not significant
**Hypotheses**			
**H0:** There is no significant difference between the groups ($p > 0.05$)			
**H1:** There is significant difference between the groups ($p < 0.05$)			

Supplementary Table S9. Statistic data from Quantification YFV immunomarking in Figure 3A.

Kruskall-Wallis test (Multiple comparisons) - *Alouatta* sp.					
**Kruskall-Wallis test**					
($\chi^2(2) = 14.77$; $p = 0.0006212$)					
**Dunn's test with Bonferroni correction**					
**chi2**	**Z**	**altP**	**P. adjusted**	**Comparisons (Regions)**	**Significance**
14.77	-3.775	0.0001599	0.0004797	M vs PC	*** (Extremely)
14.77	-2.510	0.0120900	0.0362701	M vs PV	* (Significant)
14.77	1.266	0.2056335	0.6169005	PC vs PV	ns

Kruskall-Wallis test (Multiple comparisons) - *Callithrix* sp.					
**Kruskall-Wallis test**					
($\chi^2(2) = 5.86$; $p = 0.0534$)					
**Dunn's test with Bonferroni correction**					
**chi2**	**Z**	**altP**	**P. adjusted**	**Comparisons (Regions)**	**Significance**
5,861	-21,802	0.02925	0.08774	M vs PC	ns
5,861	-0.1787	0.85817	100	M vs PV	ns
5,861	20,015	0.04534	0.13603	PC vs PV	ns

Legend: * = p<0.05 (significant); ** = p<0.005 (very significant); *** = p<0.0005 (extremely significant); **ns** (non significant).

Supplementary Table S10. Quantification of immunolabeling detection by IHC for YFV antigen, cytokines, and caspase-3 in meningeal area.

Group	Monkey ID	IHC quantification in Meninges										
		**YFV Ag**	**TNF-α**	**IL-1β**	**IFN-γ**	**IL-12**	**IFN-β**	**TGF-β**	**IL-4**	**IL-10**	**IL-13**	**Casp-3**
Alouatta sp.	476/15	33	32	48	16	32	32	16	16	32	48	32
	506/15	0	48	32	0	16	32	32	16	48	48	48
	479/16	0	48	32	16	16	16	32	32	48	64	32
	090/17	0	64	48	16	32	32	32	16	48	32	48
	273/17	0	48	48	16	32	0	16	32	16	48	48
	397/17	38	32	48	16	16	0	32	16	0	32	8
	568/17	0	64	32	16	16	16	32	16	32	48	32
	Average	0	48	41.14	13.7	22.9	18.29	27.4	20.6	32	45.71	35.43
Callitrhix sp.	815/16	66	48	48	0	16	0	32	32	32	64	16
	907/16	99	64	32	0	32	0	32	32	16	80	16
	093/17	33	64	48	16	16	16	48	32	32	48	48
	100/17	55	80	48	16	32	32	32	32	48	48	16
	103/17	0	64	32	32	16	16	32	16	32	48	48
	Average	55	64	41.6	12.8	22.4	12.8	35.2	28.8	32	57.6	28.8

* The data represents the mean quantification of each immunolabeling across three randomly selected microscopic fields.

Supplementary Table S11. Quantification of immunolabeling detection by IHC for YFV antigen, cytokines, and caspase-3 in parenchyma region.

Group	Monkey ID	IHC quantification in Parenchyma										
		**YFV Ag**	**TNF-α**	**IL-1β**	**IFN-γ**	**IL-12**	**IFN-β**	**TGF-β**	**IL-4**	**IL-10**	**IL-13**	**Casp-3**
Alouatta sp.	476/15	59,6	80	80	32	80	32	80	48	96	144	96
	506/15	71	112	96	32	64	64	96	64	112	160	80
	479/16	99	96	96	32	64	32	96	32	112	112	80
	090/17	80,6	128	128	0	80	48	64	32	128	128	96
	273/17	76	144	112	48	48	16	80	64	32	128	64
	397/17	54	112	144	32	64	32	64	80	80	96	80
	568/17	116	144	80	16	32	48	96	64	80	128	96
	Average	78,3	116,57	105,14	27,4	61,7	38,86	82,3	54,9	91,4	128	84,6
Callitrhix sp.	815/16	130	112	112	16	48	32	80	32	64	112	80
	907/16	124	128	128	48	64	48	112	64	48	144	80
	093/17	153	112	112	32	48	48	80	144	96	128	96
	100/17	134	160	112	48	96	16	80	48	112	144	64
	103/17	49	128	128	16	80	48	48	64	96	128	96
	568/17	130	128	118,4	32	67,2	38,4	80	70,4	83,2	131,2	83,2

* The data represents the mean quantification of each immunolabeling across three randomly selected microscopic fields.

Supplementary Table S12. Quantification of immunolabeling detection by IHC for YFV antigen, cytokines, and caspase-3 in perivascular region.

Groups	Monkey ID	IHC quantification in perivascular region										
		**YFV Ag**	**TNF-α**	**IL-1β**	**IFN-γ**	**IL-12**	**IFN-β**	**TGF-β**	**IL-4**	**IL-10**	**IL-13**	**Casp-3**
Alouatta sp.	476/15	60	48	32	16	32	48	32	28.8	48	80	48
	506/15	92	64	48	0	16	16	48	32	32	112	32
	479/16	99	64	64	0	16	32	48	32	32	48	32
	090/17	82,3	96	64	0	32	32	48	32	64	80	48
	273/17	100	80	64	16	48	16	32	32	16	80	32
	397/17	50	80	80	16	32	16	32	16	48	48	16
	568/17	93	80	48	16	16	32	48	32	48	64	32
	Average	92,5	73.14	57.14	9.1	27.4	27.43	41.1	32	41.1	73.14	34.3
Callitrhix sp.	815/16	123	64	64	32	32	48	32	48	32	80	16
	907/16	105	48	48	0	16	0	48	16	16	48	32
	093/17	180	80	48	16	16	16	32	32	48	80	32
	100/17	157	80	48	16	48	32	32	16	48	48	32
	103/17	50	48	64	32	16	0	16	48	32	80	32
	568/17	123	64	54.4	19.2	25.6	19.20	32	32	35.2	67.2	28.8

* The data represents the mean quantification of each immunolabeling across three randomly selected microscopic fields.

Supplementary Figure S1. Immunohistochemical staining showing the main cytokines detected in the brain of *Alouatta* sp. The arrowheads indicate cytokine immunolabeling in each cell or interstitial area. Magnification ×100 and scale bar 25 µm.

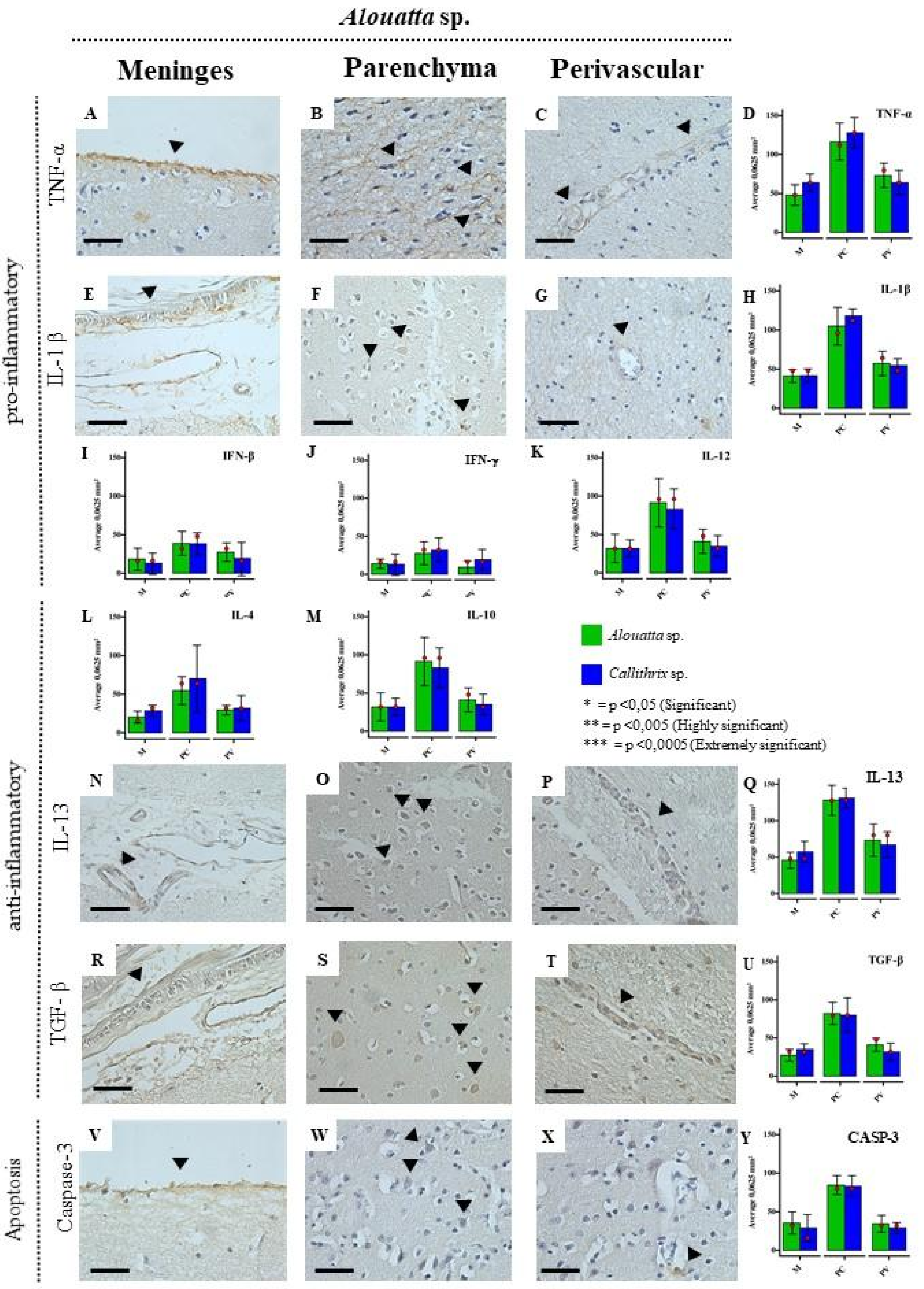

Supplement: Supplementary file 1 [file viruses-17-00386-s001.zip › viruses-3412582-supplementary.pdf]
